# Impact of the Timing of Integrated Home Palliative Care Enrolment on Emergency Department Visits

**DOI:** 10.34172/ijhpm.2022.5783

**Published:** 2022-05-17

**Authors:** Alessandro Scacchi, Armando Savatteri, Gianfranco Politano, Alessio Conti, Marco Dalmasso, Sara Campagna, Maria Michela Gianino

**Affiliations:** ^1^Department of Public Health and Paediatrics, University of Turin, Torino, Italy.; ^2^Department of Control and Computer Engineering, Politecnico di Torino, Torino, Italy.; ^3^Epidemiology Unit, Local Health Unit TO3, Grugliasco, Italy.

**Keywords:** Palliative Care, Home Care, Emergency Department Use, Timing of Care

## Abstract

**Background: ** The association between timing of integrated home palliative care (IHPC) enrolment and emergency department (ED) visits is still under debate, and no studies investigated the effect of the timing of IHPC enrolment on ED visits, according to their level of emergency. This study aimed to investigate the impact of the timing of IHPC enrolment on different acuity ED visits.

**Methods: ** A retrospective, pre-/post-intervention study was conducted from 2013 to 2019 in Italy. Analyses were stratified by IHPC duration (short ≤30 days; medium 31-90 days; long >90 days) and triage tags (white/green: low level of emergency visit; yellow/red: medium-to-high level). The impact of the timing of IHPC enrolment was evaluated in two ways: incidence rate ratios (IRRs) of ED visits were determined (1) before and after IHPC enrolment in each group and (2) post-IHPC among groups.

**Results: ** A cohort of 17 983 patients was analysed. Patients enrolled early in the IHPC programme had a significantly lower incidence rate of ED visits than the pre-enrolment period (IRR=0.65). The incidence rates of white/green and yellow/red ED visits were significantly lower post-IHPC enrolment for patients enrolled early (IRR=0.63 and 0.67, respectively). All results were statistically significant (*P*<.001). Comparing the IHPC groups after enrolment versus the short group, medium and long IHPC groups had a significant reduction of ED visits (IRR=0.37, IRR=0.14 respectively), showing a relation between the timing of IHPC enrolment and the incidence of ED visits. A similar trend was observed after accounting for triage tags of ED visits.

**Conclusion: ** The timing of IHPC enrolment is related with a variation of the incidence of ED visits. Early IHPC enrolment is related to a high significant reduction of ED visits when compared to the 90-day pre-IHPC enrolment period and to late IHPC enrolment, accounting for both low-level and medium-to-high level emergency ED visits.

## Background

**Key Messages**

** Implications for policy makers**

- Early implementation of integrated home palliative care (IHPC) programmes may effectively reduce emergency department (ED) service use while still addressing the health needs of severely ill patients towards the end of their lives.
- Evidence that patients enrolled earlier made significantly fewer visits to ED post-IHPC enrolment compared to pre-IHPC enrolment may offer policy-makers and integrated home-care service providers a valuable perspective to improve early access to palliative care for all patients with severe disabilities through the strengthening of palliative care networks.
- The results, showing that earlier IHPC enrolment is associated with reduced overall ED use, recommend to policy-makers that early enrolment in IHPC is a goal that public healthcare systems must achieve to guarantee and improve the benefits of such programmes.

** Implications for the public**

Our results found that the timing of integrated home palliative care (IHPC) had a significant relation with the incidence of emergency department (ED) visits when compared with the pre-IHPC enrolment period. Moreover, earlier IHPC enrolment is associated with reduced ED use when compared with a late enrolment. The only reduction for overall, low-level and medium-to-high level emergency visits was observed in the group who received early IHPC enrolment. Therefore, IHPC seems effective in reducing ED visits in a time-dependent manner. These results corroborate the evidence that IHPC could effectively reduce excess use of hospital resources, probably due to an efficient home-care delivery to patients, through successful management of symptoms at home.

According to the World Health Organization (WHO), “Palliative care is an approach that improves the quality of life of patients and their families facing the problems associated with life-threatening illness, through the prevention and relief of suffering by means of early identification and impeccable assessment, and treatment of pain and other problems – physical, psychosocial and spiritual.”^1^

 In Italy, palliative care is guaranteed by law (National Law 38/2010) to adult patients with cancer, severe disabilities, or progressive end-stage disorders, such as heart failure, chronic obstructive pulmonary disease, and dementia, as well as to paediatric patients with chronic and debilitating diseases.^2^ Palliative care in Italy is provided in multiple settings, including hospices, hospitals, residential facilities, and at home. Integrated home palliative care (IHPC) services are delivered and administered by teams from palliative care units. Such teams are created to handle patients’ medical, nursing, rehabilitation, and psychological needs, as well as to provide social services; their goal is to alleviate distressing symptoms to achieve the best quality of life to patients by providing complex support also during the final stages of the illness. IHPC services can also consist of the supply of medical devices for home patient management and specialist home consultations. IHPC requires a multidimensional assessment of the patient and the definition of an individual care plan.

 Evidence shows that the provision of IHPC to people at the end of their lives leads to better outcomes, such as dying at home, reducing symptoms, or even prolonging survival.^3-7^ This evidence may suggest that IHPC programmes could efficiently manage patients at home, preventing unnecessary hospital resources utilisation, such as emergency department (ED) visits. This, in turn, may result in reduced costs for public healthcare systems.

 However, there is still no agreement in the literature about the efficacy of IHPC in reducing the use of ED services. According to some reviews, there is no strong evidence of reduced ED visits among patients receiving IHPC.^3,8^ In contrast, several studies reported that IHPC had a significant impact on the decline in the average number of ED visits.^9-12^ Moreover, the association between the timing of IHPC enrolment (early or late) and ED visits is still under debate, thus little is known about the effect of the timing of IHPC enrolment on ED visits. However, several studies have shown that early enrolment in IHPC can improve many patient’s outcomes, including increased satisfaction and quality of life^13,14^ and can lead to improved survival.^15^ To date, no Italian studies have investigated ED use before and after enrolment in IHPC. Indeed, one Italian study compared patients after receiving palliative care (in home or in hospice) to patients receiving traditional care, focusing on reducing overall ED visits.^16^ In addition, no studies investigated the effect of the timing of IPHC enrolment on ED visits, based on the level of emergency. Therefore, this study aimed to investigate the impact of the timing of IHPC enrolment on different acuity ED visits.

## Methods

 A retrospective, pre-/post-intervention study was conducted on a cohort of patients enrolled in an IHPC programme between January 1, 2013 and December 31, 2019 in the Piedmont Region, with more than 4 million inhabitants over an area of 25 387 km^2^.

 Patient and ED use data were collected by merging two different sources: the Italian Official National Information System for Monitoring Home-Care Services (SIAD database) and the Italian National Information System for ED use database. These databases were merged by tracing the Universal Patient ID number, an anonymous and unique code assigned to each patient within the National Healthcare Service system fluxes and allows a suitable record linkage for epidemiological surveillances without further authorizations. Therefore, ethics committee approval was not required.

###  Intervention

 The intervention consisted of IHPC enrolment. After referral by a general practitioner, multi-professional teams decide whether a patient can receive IHPC based on specific, multi-professional assessment scales. IHPC enrolment requires creating an individual care plan, which identifies specific care goals and the most appropriate interventions in case of problems. The multi-professional team prepares the individual care plan and includes planned regular home visits and responsive day-time care. It must be shared with the patient and its family and/or caregiver. IHPC is offered for a limited and variable time duration. Upon IHPC enrolment, an electronic medical record is opened for each patient, and it is shared with the multi-professional team; this record is closed after the last service provided (eg, if a patient died or interrupted the service by being admitted to any long-term care facility). Since patients may have been enrolled in one or more IHPC programmes during the study period, to avoid potential correlation due to multiple IHPC enrolments, patients with more than one IHPC enrolment were excluded from this study.

###  Statistical Analysis

 Two time periods were compared for each patient: pre-IHPC enrolment and post-IHPC enrolment period. Pre-IHPC enrolment period took into account the 90 days prior to IHPC enrolment, and the post-IHPC enrolment period took into account the period from IHPC enrolment until the day of the last service provided. All ED visits that occurred during these periods were recorded, and incidence rates per 100 days/patient were determined for pre- and post-IHPC enrolment periods.

 The cohort of patients was divided into three groups by IHPC duration: short IHPC enrolment (duration ≤30 days), medium IHPC enrolment (duration 31-90 days) and long IHPC enrolment (duration >90 days). Patients in the long IHPC duration group (>90 days) are qualified as having early IHPC enrolment.

 First, to guarantee no confounding effect may arise due to age and sex unbalancing, the data has been pre-processed with a propensity score (PS) method implemented in R, via the WeigthIt package,^17^ separately for each IHPC duration group to form equivalent groups in the observations taking advantage of entropy balancing,^18^ which guarantees perfect balance on specified moments of the covariates while minimizing the entropy (a measure of dispersion) of the weights.

 With a balanced population we resorted to a generalized linear multivariate model with a Quasi-Poisson bias function, in order to cope with real number weights produced after PS balancing, which replaced the previous integer count figures. Although over-dispersion in the data could be captured better by a negative-binomial-based models than the plain Poisson, however, we used a Quasi-Poisson bias function since the Quasi-Poisson method adjust the Poisson method for over or under dispersion. Usually comparing a negative binomial with a Quasi-Poisson results in roughly similar results since the only difference is that the former uses weights that have a cubic relationship to the expected value whereas the latter use weights proportion linear to the expected value, so far same assumption apply to both families in term of overall data distribution. Furthermore the “rates per 100 days/patient” has been inspected with the Cullen and Frey graph and didn’t show, after PS balancing, 0s to be modelled and since the negative-binomial-based models are better in modeling the zero counts there’s probably no advantage. Eventually rates per 100 days/patient are not integer count, thus not fitting constraints of a negative binomial. Overall model for one of the analyses is: rates per 100 days/patient = BEFORE_AFTER Dummy | IHPC Duration group * Triage Tag group.

 The generalized linear model function took into account all the determinants (ie, IHPC duration group, triage tag group and a dummy variable to assess Ed visits before or after) and their interactions producing a fitted model able to predict the incidence rates per 100 days/patient. From the model so far produced, we determined the incidence rate of ED visits occurred before and after IHPC enrolment of each group by computing the estimated marginal means and their ratio computed as marginal means contrasts with a pairwise estimation corrected with Tukey *P* value for multiple comparisons.^19^ Furthermore, we applied the same approach to compare and estimate the incidence rate of ED visits among the three classes of IHPC duration within post enrolment cases.

 Statistical significance was set at *P*< .05, and 95% confidence intervals (CIs) were also reported.

 Analyses were stratified by triage tag and reason for ED visit. As defined by the D.M 15/5/92,^20^ the Italian triage system is a standardised procedure for an initial assessment in the ED. This is understood as the first classification of newly arriving patients and is meant to provide a rapid determination of safe and comprehensible treatment priorities. Triage tags are colour coded: white tag defines non-critically ill patients who do not require treatment in a short time; green tag defines borderline critically ill patients, whose intervention can be postponed; yellow tag defines medium critically ill patients, whose intervention cannot be postponed; red tag defines highly critically ill patients that need immediate emergency intervention. Thus, white and green triage tags correspond to a low level of emergency; yellow and red and triage tags correspond to a medium and high level of emergency, respectively. We grouped ED visits into white/green triage tags visits and yellow/red triage tags visits in order to highlight whether the timing of IHPC enrolment differently impacted ED visits, according to their level of emergency.

 The reason for ED visit consisted of the main symptom diagnosed during triage examination for each patient. Eight main symptoms were diagnosed: abdominal pain, dyspnoea, trauma, temperature, rhythm alteration, urological symptoms, neurological symptoms, and other symptoms. All analyses were carried out using software R version 3.6.1.

## Results

###  Patient Characteristics

 In total, 17 983 patients were enrolled in the IHPC during the study period (2013-2019). The median age was 77, and the majority of the cohort consisted of male patients (10 074; 56%). The median IHPC duration was 27 days, with an interquartile range of 10 to 64 days. Most patients were assigned to the short IHPC group (9610; 53.4%). The majority of patients (15 517; 86.3%) had a diagnosis of cancer, while 2466 (13.7%) had other chronic diseases, especially cardiovascular diseases (268; 1.5%) and neurological (253; 1.4%) (Table 1).

**Table 1:** Patients’ Characteristics

**Variables**	**No. (%) or ** **Median [IQR]**
Number of patients	17 983
Female	7909 (44)
Age	77 [68–83]
IHPC duration	27 [10–64]
IHPC duration group	
Short (<30 days)	9610 (53.5)
Medium (31-90 days)	5347 (29.7)
Long (>90 days)	3026 (16.8)
Prevalent disorder at the first evaluation in IHPC	
Cancer	15 517 (86.3)
Cardiovascular diseases	268 (1.5)
Neurological disorders	253 (1.4)
Digestive system diseases	213 (1.2)
Respiratory diseases	139 (0.8)
Urogenital diseases	99 (0.5)
Endocrine and metabolic diseases	84 (0.5)
Hematological diseases	69 (0.4)
Mental disorders	67 (0.4)
Trauma	50 (0.3)
Infectious disorders	40 (0.2)
Musculoskeletal diseases	39 (0.2)
Perinatal diseases	31 (0.2)
Missing	912 (5.1)
Other	202 (1.1)

Abbreviations: IQR, interquartile range; IHPC, integrated home palliative care.

###  Impact of the Timing of IHPC Enrolment: Analysis of ED Visits Rate Pre-Post IHPC Enrolment

 The incidence rate for overall ED visits post-IHPC enrolment was significantly higher than that observed pre-IHPC enrolment for patients enrolled later in IHPC (incidence rate ratio [IRR] = 4.35, *P* < .001, IRR = 1.61, *P* < .001 for short and medium IHPC duration, respectively) while it was significantly lower than that observed pre-IHPC enrolment for patients enrolled early in IHPC (IRR = 0.65, *P* < .001) (Table 2).

**Table 2:** IRR of ED Visits Pre- and Post-IHPC Enrolment by IHPC Duration Groups and by Triage Tag

**IHPC Duration Group**	**ED Visits Per 100 Days/Patient Pre-IHPC Enrolment**	**ED Visits Per 100 Days/Patient Post-IHPC Enrolment**	**IRR**	**95% CI**	***P *** **Value**
Short (≤30 days)	1.55	6.64	4.35	4.17-4.55	<.001
White/green ED visit	1.88	6.81	3.57	3.58-3.70	<.001
Yellow/red ED visit	1.28	6.49	5.00	5.00-5.70	<.001
Medium (31-90 days)	1.52	2.45	1.61	1.56-1.67	<.001
White/green ED visit	1.82	2.69	1.47	1.43-1.52	<.001
Yellow/red ED visit	1.26	2.23	1.75	1.67-1.89	<.001
Long (>90 days)	1.47	0.95	0.65	0.61-0.68	<.001
White/green ED visit	1.78	1.12	0.63	0.60-0.66	<.001
Yellow/red ED visit	1.21	0.80	0.67	0.60-0.74	<.001

Abbreviations: IHPC, integrated home palliative care; ED, emergency department; IRR, incidence rate ratio; CI, confidence interval. Triage tags are colour codes: white defines non-critically ill patients who do not require treatment in a short time; green defines borderline critically ill patients, whose intervention can be postponed; yellow defines medium critically ill patients, whose intervention cannot be postponed; and red defines highly critically ill patients that need immediate emergency intervention. White/Green = low level of emergency; Yellow/Red = medium-to-high level of emergency.

 Regarding triage tags, the incidence rate of white/green ED visits post-IHPC enrolment was significantly lower than that pre-IHPC enrolment for patients enrolled early (IRR= 0.63, *P* < .001), while it was higher for patients enrolled later in IHPC (IRR = 3.57, *P* < .0001, IRR = 1.47, *P* < .001, for short and medium IHPC duration, respectively). The incidence rate of yellow/red ED visits was significantly higher post-IHPC enrolment for patients enrolled later (IRR = 5.00, *P* < .001; IRR = 1.75, *P* < .001 for short and medium IHPC duration, respectively), while it was lower for patients enrolled early (IRR = 0.67, *P* < .001) in IHPC (Table 2). The incidence rate of reasons for ED visits post-IHPC enrolment for all symptoms were significantly higher for patients enrolled later than that observed pre-IHPC enrolment. Conversely, the incidence rate of reasons for ED visits post-IHPC enrolment for all symptoms were significantly lower (except for rhythm alteration) for patients enrolled earlier than that observed pre-IHPC enrolment (Table 3).

**Table 3:** ED Visits Per 100 Days/Patient Pre/Post IHPC Enrolment and IRR by IHPC Duration Group and by Reason of ED Visit

**Reason for ED visits**	**ED Visits Per 100 Days/Patient Pre-IHPC Enrolment**	**ED Visits Per 100 Days/Patient Post-IHPC Enrolment**	**IRR**	**95% CI**	***P *** **Value**
Neurological					
Short (≤30 days)	1.50	6.22	4.17	3.70-4.55	<.001
Medium (31-90 days)	1.49	2.31	1.56	1.35-1.79	<.001
Long (>90 days)	1.47	0.95	0.65	0.52-0.80	<.001
Abdominal pain					
Short	1.72	6.97	4.00	3.85-4.35	<.001
Medium	1.79	2.48	1.39	1.27-1.52	<.001
Long	1.70	1.07	0.63	0.54-0.74	<.001
Dyspnoea					
Short	1.62	6.66	4.17	3.85-4.35	<.001
Medium	1.57	2.49	1.59	1.45-1.72	<.001
Long	1.55	0.96	0.62	0.54-0.72	<.001
Trauma					
Short	1.69	6.73	4.00	3.70-4.35	<.001
Medium	1.63	2.34	1.43	1.28-1.59	<.001
Long	1.64	0.92	0.56	0.48-0.66	<.001
Temperature					
Short	1.66	6.77	4.17	3.45-4.76	<.001
Medium	1.53	2.82	1.85	1.52-2.27	<.001
Long	1.47	0.90	0.61	0.45-0.83	.002
Rhythm alteration					
Short	1.69	7.50	4.35	3.57-5.56	<.001
Medium	1.72	2.50	1.45	1.06-2.00	.018
Long	1.45	1.14	0.79	0.52-1.20	.266
Urological					
Short	2.05	7.01	3.45	3.13-3.70	<.001
Medium	2.27	2.84	1.25	1.09-1.43	.001
Long	1.83	1.34	0.73	0.59-0.91	.005
Other symptoms					
Short	1.77	6.59	3.70	3.57-3.85	<.001
Medium	1.70	2.56	1.49	1.45-1.56	<.001
Long	1.66	1.04	0.63	0.59-0.67	<.001

Abbreviations: IHPC, integrated home palliative care; ED, emergency department; IRR, incidence rate ratio; CI, confidence interval.

###  Impact of Timing of IHPC Enrolment: Analysis of ED Visits Rate Post-IHPC Enrolment in Long and Medium Versus Short IHPC Duration Group

 Incidence rates of ED visits per 100 days/patient post-IHPC enrolment declined from the short to the long IHPC duration group (from 6.64 to 0.95), as shown in Table 4. Overall, there was a reduction of the incidence of ED visits by 86% (IRR = 0.14, *P* < .001) in the long group and by 63% (IRR = 0.37, *P* < .001) in the medium group. A similar trend was observed, accounting for the triage tag of ED visits. White/green ED visits incidences reduced by 60% (IRR = 0.40, *P* < .001) in the medium group and by 84% (IRR = 0.16, *P* < .001) in the long group. However, the largest decrease affected the Yellow/Red ED visits; indeed, there was a reduction of ED visits incidences by 66% (IRR = 0.34, *P* < .001) in the medium group and by 88% (IRR = 0.12, *P* < .001) in the long group.

 The incidence rate of all reasons for ED visits post-IHPC enrolment was significantly lower for the long group compared to the short group (Table 5).

**Table 4:** ED Visits Post-IHPC Enrolment Per 100 Days/Patient and IRR in Medium, Long Versus Short IHPC Duration Group

**IHPC Duration Group and ED Visit Triage Tags**	**ED Visits Per 100 Days/Patient Post-IHPC Enrolment**	**IRR**	**95% CI**	***P *** **value**
White/green ED visits				
Short (≤30 days)	6.81	Ref	-	-
Medium (31-90 days)	2.69	0.40	0.38-0.41	<.001
Long (>91 days)	1.12	0.16	0.15-0.17	<.001
Yellow/red ED visits				
Short (≤30 days)	6.49	Ref	-	-
Medium (31-90 days)	2.23	0.34	0.30-0.36	<.001
Long (>91 days)	0.80	0.12	0.11-0.14	<.001
Total ED visits				
Short (≤30 days)	6.64	Ref	-	-
Medium (31-90 days)	2.45	0.37	0.36-0.38	<.001
Long (>91 days)	0.95	0.14	0.13-0.15	<.001

Abbreviations: IHPC, integrated home palliative care; ED, emergency department; IRR, incidence rate ratio; CI, confidence interval.

**Table 5:** ED Visits Per 100 Days/Patient Post-IHPC Enrolment and IRR by Reason of ED Visit in Medium, Long Versus Short IHPC Duration Group

**Reason for ED Visits**	**ED Visits Per 100** **Days/Patient Post-IHPC Enrolment**	**IRR**	**95% CI**	***P *** **Value**
Neurological				
Short	6.22	Ref	-	-
Medium	2.31	0.37	0.31-0.43	<.001
Long	0.95	0.15	0.12-0.19	<.001
Abdominal pain				
Short	6.97	Ref	-	-
Medium	2.48	0.35	0.31-0.40	<.001
Long	1.07	0.15	0.13-0.19	<.001
Dyspnoea				
Short	6.66	Ref	-	-
Medium	2.49	0.37	0.34-0.41	<.001
Long	0.96	0.14	0.12-0.17	<.001
Trauma				
Short	6.73	Ref	-	-
Medium	2.34	0.35	0.31-0.40	<.001
Long	0.92	0.14	0.12-0.16	<.001
Temperature				
Short	6.77	Ref	-	-
Medium	2.82	0.42	0.33-0.53	<.001
Long	0.90	0.13	0.09-0.18	<.001
Rhythm alteration				
Short	7.50	Ref	-	-
Medium	2.50	0.33	0.23-0.46	<.001
Long	1.14	0.15	0.10-0.23	<.001
Urological				
Short	7.01	Ref	-	-
Medium	2.84	0.41	0.35-0.48	<.001
Long	1.34	0.19	0.15-0.24	<.001
Other symptoms				
Short	6.59	Ref	-	-
Medium	2.56	0.39	0.37-0.41	<.001
Long	1.04	0.16	0.15-0.17	<.001

Abbreviation: ED, emergency department; IHPC, integrated home palliative care; IRR, incidence rate ratio; CI, confidence interval.

## Discussion

 This study aimed to investigate whether the timing of IHPC enrolment affected ED visits in a large sample of Italian patients. Our results reported an association between the timing of IHPC enrolment and the incidence of the ED visits, when compared with the pre-IHPC enrolment period, in the long IHPC duration group. Moreover, earlier IHPC enrolment is associated with reduced ED use when compared with a late enrolment. These achievements corroborate the suggestion that IHPC may effectively reduce ED visits in a time-dependent manner. When patients received IHPC for ≤30 days (short IHPC duration group), there was an overall increase of ED visits in comparison with pre-IHPC enrolment. This also happened for patients who received IHPC for 3 months (medium IHPC duration group), even if the increase was lower than that observed in the short IHPC duration group. On the contrary, when a patient received IHPC for more than 3 months (long IHPC duration group), there was a strong overall reduction in ED visits, supporting the hypothesis that IHPC tended to prevent them. In addition, the effect of the timing of IHPC enrolment on the incidence rate of ED visits may be hypothesized when comparing different IHPC enrolment groups. Indeed, the IRR of ED visits showed a decline from short to long IHPC duration group post-IHPC enrolment.

 Some previous studies have already shown a significant decline in the average number of ED visits after IHPC enrolment as compared to the period before enrolment. American studies, analysing electronic medical records, reported that there was a significant decline in the average number of ED visits after enrolment in a home-based palliative care program compared to the period before (average: 1 versus 1.79, *P* > .001)^11^ and that there was an overall reduction in ED visits by 25% among home palliative care recipients in the 12 months before and following enrollment.^21^ Based on administrative data, Swedish research showed that patients who were alive 90 days after admission to a home palliative care program had a reduction by 51% of ED visits (from 2188 to 1071) compared to the 90 days before admission.^22^

 Other previous studies demonstrated that early IHPC enrolment is associated with reduced use of ED services. A Canadian study reported that between those who received early palliative care (from 12 to 6 months before death), compared with a control group of not-early palliative care recipients, there was a lower proportion of whom used ED visits (40% vs 50.1%; *P* = .001) in the last month of life.^23^ An Australian study^24^ estimated that an earlier admission to home palliative care (over 6 months before death) was associated with a lower rate of ED visits. In Ontario, Seow showed a significant association between admission time and the OR of having an ED visit in a time-response manner: the OR of having an ER visit in the 2 weeks before death reduces from 20% (for an admission time from 5 to 12 weeks before death) to 29% (for admission time more than 24 weeks) in comparison with patients enrolled from 3 to 4 weeks before death.^25^ In the United States, Bevins et al reported that patients receiving early palliative care (within 30 days from the diagnosis of pancreatic cancer) had fewer ED visits (2.65 vs. 2.81 visits, *P* = .001) compared with patients receiving late palliative care.^26^

 While consistent with the literature, our study adds that the enrolment in a palliative care program is not the only factor related to the change in the rate of ED visits, but also the timing of IHPC enrolment (a long IHPC duration) has a significant relation with the incidence of the ED visits, when compared with the pre-IHPC enrolment period. In addition, our findings are original given that we also explored the impact of the timing of IHPC on ED visits, based on their emergency acuity. Indeed, our results showed that the long IHPC duration group had a decreased IRR of both low-level and medium-to-high level of emergency ED visits, whereas the IRR of ED visits tended to increase in patients enrolled later. Therefore, early IHPC enrolment showed a significant relation with the reduction of medium-to-high level emergency ED visits compared with the 90-day pre-IHPC enrolment period, with a steeper negative slope than low-level emergency ED visits.

 Several explanations may be brought forward for these original findings. The long IHPC duration may offer more appropriate and adequate attention towards managing complex symptoms, thus preventing high acuity issues from occurring. Indeed, IHPC teams work with a comprehensive approach, which includes interaction with other healthcare professionals to provide optimized disease-specific care. While carrying out their IHPC tasks, these teams might successfully manage different symptoms, such as abdominal pain and dyspnoea, before they exacerbate. Moreover, it is possible that early IHPC enrolment allows providers a prompt identification and anticipation of some patient needs directly at home and thereby it might prevent new problems from arising, avoiding the need for acute care later on.^25,27^ These suggestions are corroborated by another of our results, which highlighted the decrease of ED visits for all reasons among patients receiving IHPC for more than 3 months, namely those enrolled earlier.

 Our results may also be explained by considering that long IHPC duration can guarantee prolonged support by the palliative team, providing family members with the skill set necessary to manage an illness and offering emotional and advocacy support as needed, to help prevent caregiver burnout. In agreement with other studies, this support can help the family members more consciously assess when it is appropriate to visit ED or stay at home, even when patients develop high-acuity issues.^28,29^

 Altogether, better symptoms relief, easier access to identify and anticipate the potential risks of adverse outcomes, and counselling and supporting family members may explain the reduced need for emergency care, especially those at low-level of emergency. Early IHPC enrolment would seem to meet better the WHO’s definition of palliative care, whose goal is achieved by prevention and relief of suffering through early identification, flawless assessment and treatment of pain and other problems - physical, psychosocial and spiritual.^1^

 To our knowledge, this is the first work that investigated the impact of the timing of IHPC enrolment on ED visits, accounting also for their level of emergency.

 One of the limitations of this study is that the timing of enrolment can be defined as early or late only after each closure of an IHPC medical record, making it harder to suggest strategies for timely caring for current or future patients. However, the large and representative study population acted as an advantage, making the research broadly applicable (particularly to other European countries with a similar population health profile and organisation of community-based home-care services), suggesting the strategy that early enrolment in IHPC is a goal that public healthcare systems must achieve in order to obtain similar positive results from these programs. Other major limitations of the study are those regarding the information sources used and are common to all administrative database studies. These include problems related to the quality of data collection, especially the possible lack of accuracy and different coding criteria used by individuals and across different institutions. Moreover, some variables can affect the amount of ED visits, such as symptom severity, the quality of delivered care, or the use of privately obtained services that are not recorded in administrative databases. By contrast, and as demonstrated by the majority of published literature, these databases are the best available for large-scale epidemiological studies and monitoring population trends in service utilisation.

## Conclusion

 The timing of IHPC enrolment is related to a variation of ED visits incidence. Early IHPC enrolment resulted in a significant reduction of ED visits compared with the 90-day pre-IHPC enrolment period and with late IHPC enrolment, accounting for both low-level and medium-to-high level emergency ED visits. These results may offer policy-makers and integrated home-care service providers a valuable perspective to improve access to palliative care for all patients with severe disabilities through the strengthening of palliative care networks.Other considerations, such as patients’ preference or caregiver presence, are essential but will require alternative study designs.

## Ethical issues

 Data about patients, anonymised by the Universal Patient ID Number, are available for use in administrative and/or epidemiological studies without further authorisation. Therefore, ethics committee approval was not required for this study.

## Competing interests

 Authors declare that they have no competing interests.

## Authors’ contributions

 MMG conceptualized and designed the study, AS and MMG drafted the initial manuscript, and reviewed and revised the manuscript. MD designed the data collection instruments, collected data. GP carried out the analyses. SC, ArS, MMG, and AC critically reviewed the manuscript for important intellectual content. All authors approved the final manuscript as submitted and agree to be accountable for all aspects of the work.

## Funding

 This research received no specific grant from any funding agency in the public, commercial, or not for-profit sectors.
